# Effect of Marine Polyunsaturated Fatty Acids on Biofilm Formation of *Candida albicans* and *Candida dubliniensis*

**DOI:** 10.3390/md8102597

**Published:** 2010-10-08

**Authors:** Vuyisile S. Thibane, Johan L. F. Kock, Ruan Ells, Pieter W. J. van Wyk, Carolina H. Pohl

**Affiliations:** 1 Department of Microbial, Biochemical and Food Biotechnology, University of the Free State, P.O. Box 339, Bloemfontein, 9301, South Africa; E-Mails: ThibaneV@ufs.ac.za (V.S.T.); KockJL@ufs.ac.za (J.L.F.K.); EllsR@ufs.ac.za (R.E.); 2 Center for Microscopy, University of the Free State, P.O. Box 339, Bloemfontein, 9301, South Africa; E-Mail: vanWykPW@ufs.ac.za

**Keywords:** Candida albicans, Candida dubliniensis, polyunsaturated fatty acids

## Abstract

The effect of marine polyunsaturated fatty acids on biofilm formation by the human pathogens *Candida albicans* and *Candida dubliniensis* was investigated. It was found that stearidonic acid (18:4 n-3), eicosapentaenoic acid (20:5 n-3), docosapentaenoic acid (22:5 n-3) and docosahexaenoic acid (22:6 n-3) have an inhibitory effect on mitochondrial metabolism of both *C. albicans* and *C. dubliniensis* and that the production of biofilm biomass by *C. dubliniensis* was more susceptible to these fatty acids than *C. albicans*. Ultrastructural differences, which may be due to increased oxidative stress, were observed between treated and untreated cells of *C. albicans* and *C. dubliniensis* with formation of rough cell walls by both species and fibrillar structures in *C. dubliniensis*. These results indicate that marine polyunsaturated fatty acids may be useful in the treatment and/or prevention of biofilms formed by these pathogenic yeasts.

## 1. Introduction

*Candida albicans* and *Candida dubliniensis* are dimorphic yeasts, able to grow both as yeasts and mycelia. Several members of the genus *Candida* exist as commensals of the human gastrointestinal and genitourinary tract in healthy individuals [1–3]. However, in individuals whose immune system is compromised, such as those that are HIV positive, *C. albicans* can cause diseases ranging from superficial infections to deep seated mycoses [4,5]. *Candida dubliniensis* is a species closely related to *C. albicans* and a causative agent of oropharyngeal candidiasis in immunocompromised humans [5,6]. Biofilm formation is a major virulence factor in the pathogenicity of *Candida* species, partly due to their increased resistance to antifungal treatment [7,8]. Since biofilm associated infections have many clinical and economic consequences, recent research into the pathogenicity of *Candida* species has focused on the prevention and management of these biofilms. Fatty acids have been known to have antibacterial [9] and antifungal properties and especially capric acid (10:0) and lauric acid (12:0) are known to have anti-*Candida* effects by inhibiting growth of planktonic cells [10] and butyric acid (4:0) was shown to inhibit hyphal formation by *C. albicans* [11].

Marine lipids, such as fish oils, are well known sources of medium to long chain polyunsaturated fatty acids (PUFAs) and are enriched in n-3 PUFAs [*i.e.*, stearidonic acid (18:4 n-3), eicosapentaenoic acid (20:5 n-3), docosapentaenoic acid (22:5 n-3) and docosahexaenoic acid (22:6 n-3)] [12]. In addition, marine algae provide alternative sources of these n-3 PUFAs [13]. The lipid compositions and quantity of marine lipids vary depending on species and climatic conditions, with lipids from organisms in colder regions having a higher PUFA content than those in warmer regions [14,15]. Although some marine PUFAs have beneficial effects in several human diseases [16–18], the effectiveness of marine PUFAs against *Candida* biofilms have not been assessed previously. Therefore, the aim of this study was to determine the effect of long chain marine PUFAs [18:4 n-3, arachidonic acid (20:4 n-6), 20:5 n-3, 22:5 n-3 and 22:6 n-3] on biofilm formation by *C. albicans* and *C. dubliniensis*.

## 2. Results and Discussion

### 2.1. Inhibition of mitochondrial metabolism

**Figure 1:**
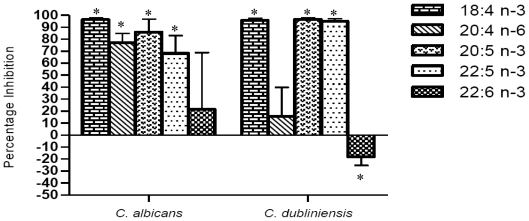
Effect of marine fatty acids (18:4 n-3, 20:4 n-3, 20:5 n-3, 22:5 n-3, 22:6 n-3) on mitochondrial metabolism of *C. albicans* and *C. dubliniensis* biofilms. Biofilms were grown in the presence of 1 mM of the fatty acids and mitochondrial activity was monitored using the XTT assay. The percentage inhibition values were determined compared to untreated controls. n = 8; * significantly different from control (*P* ≤ 0.01).

As indicated in Figure 1 the mitochondrial metabolism of biofilms of *C. albicans* and *C. dubliniensis*, assessed by XTT assay, was significantly inhibited by 1 mM 18:4 n-3, 20:5 n-3 and 22:5 n-3. In addition, biofilms of *C. albicans* were also sensitive to 20:4 n-6, while biofilms of *C. dubliniensis* were not significantly inhibited. Interestingly, 22:6 n-3 did not significantly inhibit mitochondrial metabolism of *C. albicans* or *C. dubliniensis* biofilms. As a result 20:4 n-6 and 22:6 n-3 were not used in further experiments.

### 2.2. Inhibition of biomass production

**Figure 2:**
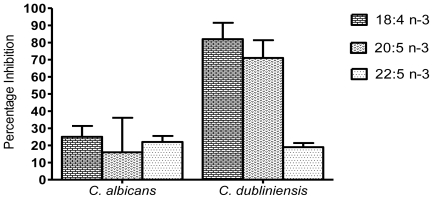
Inhibition of biofilm biomass of *C. albicans* and *C. dubliniensis* compared to untreated controls. Biofilms were grown in the presence of 1 mM of the marine PUFAs (18:4 n-3, 20:5 n-3, 22:5 n-3) and biofilm dry weight was determined on pre-weighed filters. n = 2.

Although several authors use the XTT reduction assay as an indicator of biofilm biomass, Kuhn and co-workers [19] has cautioned against this approach. Therefore, the effect of the marine PUFAs on biofilm biomass production was determined by dry weight. As indicated in Figure 2, biofilm biomass production by *C. dubliniensis* was susceptible to the three tested PUFAs, with 18:4 n-3 and 20:5 n-3 resulting in a reduction of *circa* 82% and 71%, respectively. *C. dubliniensis* biofilm biomass was less susceptible to 22:5 n-3, which produced an inhibition of *circa* 19%. Similar results were obtained between the two species for the XTT reduction assay, however, the biofilm biomass of *C. albicans* was generally less susceptible to the tested PUFAs and a reduction of only *circa* 25% and 22% was seen for 18:4 n-3 and 22:5 n-3, respectively. Although a *circa* 16% reduction in *C. albicans* biofilm biomass was observed with 20:5 n-3, this was not statistically significant. These results may indicate the increased ability of the *C. albicans* strain to obtain energy through pathways that do not require mitochondrial metabolism.

### 2.3. Morphology of cells in biofilms

**Figure 3:**
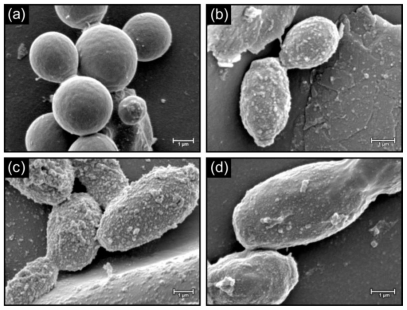
SEM micrograps showing cells of *C. albicans* control biofilms (**a**) and biofilms treated with 1 mM 18:4 n-3 (**b**), 20:5 n-3 (**c**) and 22:5 n-3 (**d**).

**Figure 4:**
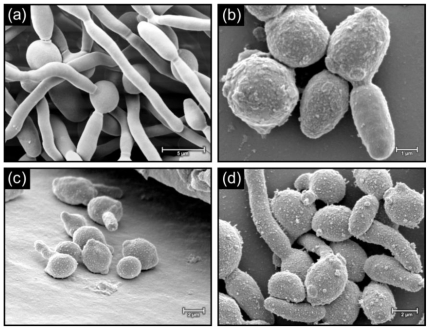
SEM micrographs showing cells of *C. dubliniensis* control biofilms (**a**) and biofilms treated with 1mM 18:4 n-3 (**b**), 20:5 n-3 (**c**) and 22:5 n-3 (**d**).

Figure 3 depicts biofilms of *C. albicans* grown in the presence and absence of 1 mM 18:4 n-3, 20:5 n-3 and 22:5 n-3. In the absence of the PUFAs, the cell surface appeared smooth (Figure 3a) and when grown in the presence of PUFAs, cells had a rough appearance with protuberances (Figure 3b, c, d). Similar results were also observed for biofilms of *C. dubliniensis* when grown in the absence (Figure 4a), and the presence (Figure 4b, c, d), of the PUFAs with protuberances and fibrillar structures visible on the cell surface. Similar rough cell surfaces were observed when *C. albicans* was exposed to miconazole [20], which is known to cause an increase in reactive oxygen species in *Candida* biofilm cells [21]. Lemar *et al.* [22] also found that *C. albicans* cells were not smooth in the presence of alyl alcohol, which increases oxidative stress. Furthermore, in a study by Leeuw *et al.* [23] on the yeast *Cryptococcus curvatus* grown on oxidised lipids, protuberances were observed on the cell surface. We therefore speculate that the changes in cell surface in the presence of PUFAs might be due to increased lipid peroxidation and resultant oxidative stress. This will be studied in future.

## 3. Experimental

### 3.1. Strains used

*Candida albicans* CBS 562T and *Candida dubliniensis* NRRL Y-17841T were used in this study and were maintained on yeast malt extract (YM) agar plates (10 g/L glucose, 3 g/L yeast extract, 3 g/L malt extract, 5 g/L peptone, 16 g/L agar) at room temperature. The strains were stored on agar slants at 4 °C.

### 3.2. XTT assay of biofilms

Cells of *C. albicans* and *C. dubliniensis* were grown separately on YM agar plates and incubated at 30 °C for 24 hours. After incubation, a loop-full of the cells was inoculated into 20 mL of yeast nitrogen base (YNB) glucose medium (10 g/L glucose, 6.7 g/L YNB) and incubated for 48 hours at 30 °C. Cells were washed three times with phosphate buffered saline (PBS) and diluted in RPMI-1640 medium (Sigma-Aldrich, UK) to an initial cell concentration of 1 × 10^6^ cells/mL. A volume of 100 μL of the standardized cell suspension was dispensed into a 96-well microtiter plate (Corning Incorporated, Costar^®^, U.S.) and incubated for 1 hour at 37 °C to allow adherence of cells to the surface [24]. Wells were washed twice with PBS to remove non-adherent cells. Mature biofilms were formed at 37 °C for 47 hours in the presence and absence of 1 mM of the marine fatty acids (18:4 n-3, 20:4 n-3, 20:5 n-3, 22:5 n-3, 22:6 n-3) (Sigma-Aldrich, UK). The reduction of (2,3-bis(2-methoxy-4-nitro-5-sulfophenyl)-5-[(phenylamino)carbonyl]-2H-tetrazolium hydroxide) (XTT) (Sigma Aldrich) was used to examine the yeast viability by measuring the mitochondrial metabolic activity of the biofilms, according to the method of Kuhn *et al.* [19]. XTT is converted to the diffusible, water soluble formazan that is colored and is easily measured in cellular supernatants in terms of optical density at 492 nm. This experiment was done in duplicate on two different days and four values obtained for each repetition (n = 8). The average and standard deviations were calculated and the student’s *t*-test was used to determine the significance of data sets.

### 3.3. Biomass determination

Twenty milliliters of a standardized cell suspension (1 × 10^6^ cells/mL), prepared as before, was inoculated into Petri dishes containing RPMI-1640 medium and allowed to adhere to the surface for 1 hour at 37 °C. Non-adherent cells were removed with PBS and mature biofilms were formed in the presence of 1 mM of the marine fatty acids (18:4 n-3, 20:5 n-3, 22:5 n-3) for 47 hours at 37 °C. Untreated biofilms served as controls. Mature biofilms were washed to remove non-adherent cells, scraped off and resuspended in PBS. Cells were filtered on pre-weighed 0.2 μm cellulose acetate filters (Lasec, SA). The filters were dried to constant weight for 48 hours at 37 °C and the biomass determined. This experiment was done in duplicate and the mean and range calculated.

### 3.4. Morphological examination

The standardized cell suspension (1 × 10^6^ cells/mL), prepared as before, was added to chamber slides (Lab-Tek^®^ Chamber Slide™ System 177372) containing silicone rubber disks (diam 5.5 mm) and 4 mL RPMI-1640 medium. Cells were allowed to adhere for 1 hour at 37 °C. Non-adherent cells were removed with PBS and mature biofilms were formed in the presence of 1 mM of the marine fatty acids (18:4 n-3, 20:5 n-3, 22:5 n-3) for 47 hours at 37 °C, with appropriate controls. The silicone rubber disks were removed and fixed for 2 hours using 3% (v/v; 1.0 M) sodium phosphate buffered glutardialdehyde, followed by fixing for 1 hour with a similarly buffered solution of osmium tetroxide (1% m/v). The disks were dehydrated in a graded series of ethanol solutions (50%, 70% and 95%) for 20 minutes and absolute ethanol for 1 hour. They were then critical-point dried, mounted and coated with gold to make them electrically conductive and finally visualized on a Shimadzu SSX550 SEM (Japan) microscope according to the method of van Wyk and Wingfield [25].

## 4. Conclusions

Certain marine PUFAs, especially 18:4 n-3, 20:5 n-3 and 22:5 n-3, have an inhibitory effect on the mitochondrial activity of both *C. albicans* and *C. dubliniensis* biofilms and significantly inhibited biofilm biomass of *C. dubliniensis.* These marine PUFAs also affected cellular morphology of biofilms of both species. This may be due to increased oxidative stress as a result of incorporation of PUFAs into the cellular lipids. These findings suggest that marine PUFAs may be useful in the treatment and/or prevention of *Candida* biofilms, which are known to have increased antifungal resistance compared to free-living cells.
